# Inhibition of IL-6 expression in LNCaP prostate cancer cells by a combination of atorvastatin and celecoxib

**DOI:** 10.3892/or.2013.2885

**Published:** 2013-11-29

**Authors:** HUAQIAN WANG, XIAO-XING CUI, SUSAN GOODIN, NING DING, JEREMIAH VAN DOREN, ZHIYUN DU, MOU-TUAN HUANG, YUE LIU, XIAODONG CHENG, ROBERT S. DIPAOLA, ALLAN H. CONNEY, XI ZHENG

**Affiliations:** 1 Allan H. Conney Laboratory for Anticancer Research, Guangdong University of Technology, Guangzhou 510006, P.R. China; 2 Department of Chemical Biology, Susan Lehman Cullman Laboratory for Cancer Research, Ernest Mario School of Pharmacy, Rutgers, The State University of New Jersey, Piscataway, NJ 08854, USA; 3 The Cancer Institute of New Jersey, New Brunswick, NJ 08903, USA; 4 School of Life Sciences and Technology, East Hospital, Tongji University, Shanghai 200092, P.R. China

**Keywords:** prostate cancer, IL-6, atorvastatin, celecoxib, xenograft tumor

## Abstract

In the present study, we investigated the effect of a combination of atorvastatin and celecoxib on the formation of interleukin (IL)-6, a cytokine that is increased during the progression of LNCaP tumors from androgen dependence to androgen independence. Culturing LNCaP cells in androgen-depleted (AD) medium increased the levels of IL-6 and survivin, and treatment of the cells in AD medium with a combination of atorvastatin and celecoxib strongly inhibited the increase in IL-6 and survivin which is one of the downstream targets of the IL-6 signaling pathway. Addition of recombinant IL-6 partially abrogated the combined effect of atorvastatin and celecoxib on apoptosis in LNCaP cells cultured in AD medium. In SCID mice, we found that the levels of IL-6 and survivin expression were increased when LNCaP tumors became androgen-independent. Treatment of the mice with atorvastatin or celecoxib alone caused decrease in the levels of IL-6 and survivin as LNCaP tumors became androgen-independent, but treatment of the mice with a combination of celecoxib and atorvastatin resulted in a much stronger inhibition in the increase in IL-6 and survivin expression. Our results indicate that decreases in IL-6 and survivin levels by atorvastatin and celecoxib administration are associated with increased apoptosis in LNCaP cells treated with this drug combination. Our *in vivo* studies indicate that the inhibitory effect of a combination of atorvastatin and celecoxib on the progression of androgen-dependent LNCaP xenograft tumors to androgen independence is associated with inhibition of the increase in IL-6 and survivin that occurs when androgen-dependent LNCaP prostate tumors become androgen-independent.

## Introduction

Advanced prostate cancer requires androgen for growth and usually responds to androgen deprivation therapy (1–4). The disease progresses, however, due to the development of mechanisms of resistance to an androgen-independent state (also referred to as castration-resistant or hormone-refractory) (5,6). Additional therapies such as chemotherapy and newer antiandrogens are only temporarily effective (7,8). Therefore, novel and less toxic approaches for delaying the progression of prostate cancer to androgen independence or delaying the need to start such additional therapies would change the treatment paradigm for managing prostate cancer and be of great benefit for patients.

Atorvastatin is a widely used statin drug for lowering cholesterol (9,10). Celecoxib is a selective cyclooxygenase-2 (COX-2) inhibitor. Previous studies investigating either statin drugs (including atorvastatin) or celecoxib have found that they have anti-prostate cancer activity (11–16). However, the effects of these two drugs in combination on prostate cancer progression to androgen independence have not yet been studied. Our previous study found that administration of a combination of atorvastatin and celecoxib was more effective than either agent alone for inhibiting azoxymethane-induced colon carcinogenesis in rats (17). More recently we found that atorvastatin and celecoxib in combination synergistically inhibited the growth and induced apoptosis in cultured prostate cancer cells. This combination inhibited the progression of androgen-dependent LNCaP tumors to androgen independence and the growth of androgen-independent PC-3 prostate tumors in SCID mice more effectively than either agent alone (18,19). Based on our preclinical studies, we have a phase II clinical trial underway to determine whether a combination of atorvastatin and celecoxib can stabilize or decrease a previously rising PSA in prostate cancer patients who developed biochemical relapse after surgery or radiation therapy. Due to the beneficial effects, the study has now been expanded into a national trial involving multiple cancer centers (NCT01220973). Although our previous *in vitro* cell culture and *in vivo* animal experiments showed that atorvastatin in combination with celecoxib inhibited androgen-independent growth of prostate cancer cells, the mechanisms for the effect are not clear. Since increased interleukin (IL)-6 has been associated with progression of androgen-dependent prostate cancer to androgen independence (20–26), we hypothesized that atorvastatin and celecoxib in combination would strongly inhibit the increased formation of IL-6 that occurs during the formation of androgen-independent LNCaP prostate tumors and that this inhibition would lead to the suppression of prostate cancer growth.

In the present study, we determined the effect of atorvastatin and celecoxib administration alone or in combination on IL-6 levels in androgen-dependent prostate cancer LNCaP cells grown in androgen-deficient medium. We found that culturing LNCaP cells in androgen-depleted (AD) medium increased the levels of IL-6 and survivin, and treatment of these cells with atorvastatin in combination with celecoxib inhibited the increase in IL-6 and survivin. In animal experiments, we found that IL-6 expression was increased in androgen-independent LNCaP tumors. Treatment of the mice with atorvastatin or celecoxib alone inhibited the increase in IL-6 and survivin as LNCaP tumors became androgen-independent, and treatment of the mice with a combination of celecoxib and atorvastatin resulted in a much stronger inhibition.

## Materials and methods

### Cells and reagents

LNCaP cells were obtained from the American Type Culture Collection (ATCC, Rockville, MD, USA). Atorvastatin and celecoxib were provided by the National Cancer Institute’s Repository. Propylene glycol, polysorbate 80, benzyl alcohol, ethanol and dimethyl sulfoxide (DMSO) were purchased from Sigma (St. Louis, MO, USA). Matrigel was obtained from BD Biosciences (Bedford, MA, USA). RPMI-1640 tissue culture medium, penicillin-streptomycin, L-glutamine and fetal bovine serum (FBS) were from Gibco-BRL (Grand Island, NY, USA). Charcoal-stripped FBS was purchased from HyClone Inc. (Logan, UT, USA). LNCaP cells were maintained in RPMI-1640 culture medium containing 10% FBS that was supplemented with penicillin (100 U/ml)-streptomycin (100 μg/ml) and L-glutamine (300 μg/ml). Atorvastatin and celecoxib were dissolved in DMSO, and the final concentration of DMSO in all experiments was 0.2%. In experiments with AD medium, charcoal-stripped FBS was used to replace the regular FBS in the cell culture medium.

### Determination of the number of viable cells

The number of viable cells after each treatment was determined using a hemacytometer under a light microscope (Nikon Optiphot, Japan). Cell viability was determined by the trypan blue exclusion assay, which was conducted by mixing 80 μl of the cell suspension and 20 μl of 0.4% trypan blue solution for 2 min. Blue cells were counted as dead cells and the cells that did not absorb dye were counted as live cells.

### Morphological assessment of apoptotic cells

Apoptosis was determined by morphological assessment of cells stained with propidium iodide (27). Briefly, cytospin slides were prepared after each experiment, and cells were fixed with acetone/methanol (1:1) for 10 min at room temperature, followed by 10 min with propidium iodide staining (1 μg/ml in PBS) and analyzed using a fluorescence microscope (Nikon Eclipse TE200, Japan). Apoptotic cells were identified by classical morphological features including nuclear condensation, cell shrinkage and formation of apoptotic bodies (27). At least 200 cells were counted in each sample, and the percentage of apoptotic cells was determined.

### Western blotting

An antibody against survivin (AB3610) was obtained from the Millipore Co. (Billerica, MA, USA). The western blot analysis was performed as described in detail in our previous publication (28). The extent of protein loading was determined by blotting for β-actin, and the levels of survivin in the western blotting were analyzed by optical density measurement and normalized for β-actin to obtain the relative density (RD) for the control and cells treated with atorvastatin and/or celecoxib.

### Progression of androgen-dependent prostate LNCaP tumors to androgen independence in immunodeficient mice

Male SCID mice with androgen-dependent LNCaP tumors were surgically castrated and injected with vehicle (5 μl/g body weight), atorvastatin (10 μg/g body weight), celecoxib (10 μg/g body weight) or atorvastatin (5 μg/g body weight) + celecoxib (5 μg/g body weight) once a day for 42 days as described in detail in our previous publication (18). The animal experiment was carried out under an Institutional Animal Care and Use Committee (IACUC)-approved protocol.

### Immunostaining

An immunoperoxidase staining method (29) was used to determine the level of IL-6 in LNCaP cells and the level of IL-6 and survivin in LNCaP tumors. Briefly, cytospin slides of LNCaP cells or paraffin sections of LNCaP tumors were incubated with either anti-IL-6 (AF-206-NA; R&D Systems, San Diego, CA, USA) or anti-survivin (AB3610) antibody for 1 h at room temperature. The sections and cytospin slides were then incubated with a biotinylated secondary antibody for 30 min followed by incubation with conjugated-avidin solution (Elite ABC kit purchased from Vector Laboratories) for 30 min. Color development was achieved by incubation with 0.02% 3,3′-diaminobenzidine tetrahydrochloride containing 0.02% hydrogen peroxide for 10 min at room temperature.

### Statistical analyses

The analysis of variance (ANOVA) method with the Tukey-Kramer test (30) was used for the comparison of growth inhibition and apoptosis. The potential synergistic effect of atorvastatin and celecoxib was assessed by the isobole method (31), using the equation Ac/Ae + Bc/Be = combination index (CI); where Ac and Bc represent the concentration of drug A and drug B used in the combination, and Ae and Be represent the concentration of drug A and B that produced the same magnitude of effect when administered alone. If CI is <1, then the drugs are considered to act synergistically. If the CI is >1 or =1, then the drugs act in an antagonistic or additive manner, respectively.

## Results

### Effects of atorvastatin and celecoxib on growth and apoptosis in cultured prostate cancer LNCaP cells

**Figure 1:**
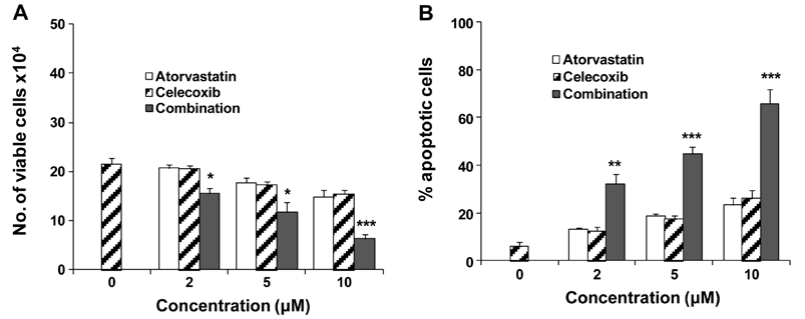
Effect of atorvastatin and/or celecoxib on the growth and apoptosis of LNCaP cells. LNCaP cells (0.5×10^5^ cells/ml) were seeded in 35-mm tissue culture dishes (2 ml/dish) and incubated for 24 h in regular medium. The cells were then cultured in androgen-depleted (AD) medium and treated with atorvastatin or celecoxib alone or in combination for 96 h. (A) The number of viable cells was determined by a trypan blue exclusion assay. (B) Apoptosis was determined by propidium iodide staining and morphological assessment. Each value is the mean ± SE from 3 experiments. Differences in the number of viable cells between a combination group and a single agent-treated group were analyzed by the Tukey-Kramer multiple comparison test. ^*^p<0.05, ^**^p<0.01, ^***^p<0.001.

We determined the effects of atorvastatin and celecoxib alone or in combination on growth and apoptosis in LNCaP cells cultured in AD medium. LNCaP cells were cultured in AD medium and treated with different concentrations of atorvastatin and celecoxib alone or in combination for 96 h. Cell growth was determined by counting the number of viable cells (Fig. 1A). As shown in Fig. 1A, treatment of LNCaP cells with atorvastatin and celecoxib in combination had a stronger inhibitory effect on cell growth than either drug alone. The CI (31) for IC_50_ was 0.81 indicating a synergistic effect when the cells were treated with a combination of atorvastatin and celecoxib. Apoptosis in LNCaP cells treated with atorvastatin and/or celecoxib was determined by morphological assessment (Fig. 1B). As shown in Fig. 1B, atorvastatin and celecoxib in combination had a stronger stimulatory effect on apoptosis than either drug alone. Indeed the apoptotic response following treatment with 2 μM of each drug in combination was greater than that for 10 μM of atorvastatin or celecoxib alone (Fig. 1B). The CI for 50% apoptosis was calculated as 0.56 indicating synergy.

### Effects of atorvastatin and celecoxib alone or in combination on the levels of IL-6 and survivin in cultured LNCaP cells

**Figure 2:**
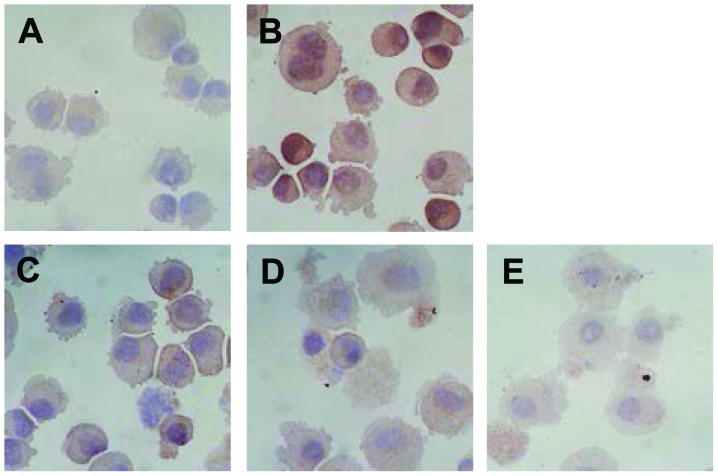
Effect of atorvastatin and/or celecoxib on IL-6 expression in LNCaP cells. LNCaP cells were seeded at a density of 0.5×10^5^ cells/ml in 35-mm tissue culture dishes (2 ml/dish) and incubated in regular medium for 24 h. The cells were then treated with atorvastatin or celecoxib alone or in combination for 24 h in androgen-depleted (AD) medium. Immunostaining with an IL-6 antibody was used to determine the expression of IL-6 in cells in regular medium (A); in AD medium (B); in AD medium and treated with 10 μM atorvastatin (C); in AD medium and treated with 10 μM celecoxib (D) and in AD medium and treated with 10 μM atorvastatin + 10 μM celecoxib (E). The data presented are representative of 3 experiments. IL-6, interleukin-6.

**Figure 3:**
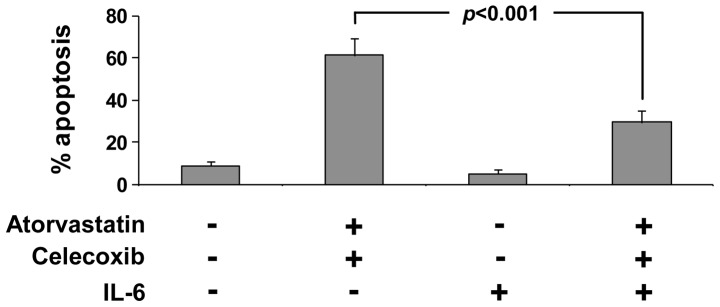
Effect of recombinant IL-6 on apoptosis in LNCaP cells treated with atorvastatin and celecoxib. LNCaP cells were seeded at a density of 0.2×10^5^ cells/ml in regular medium for 24 h. The cells were then cultured in androgen-depleted (AD) medium and treated with atorvastatin (10 μM) and celecoxib (10 μM) with/without IL-6 (50 ng/ml; #206IL) for 96 h. Apoptotic cells were determined by morphological assessment. Each value is the means ± SE from 3 experiments. Statistical analysis was carried out using ANOVA with Tukey-Kramer multiple comparison. IL-6, interleukin-6.

**Figure 4:**
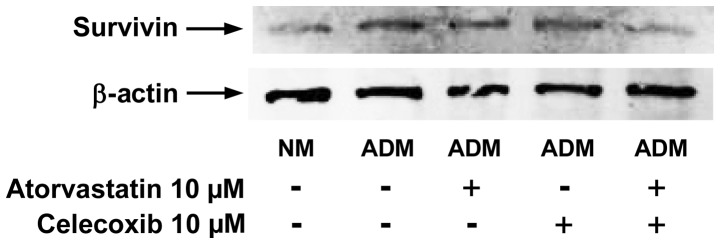
Effect of atorvastatin and/or celecoxib on the level of survivin in LNCaP cells. LNCaP cells were seeded at a density of 1×10^5^ cells/ml in regular normal medium (NM) and incubated for 24 h. The NM was then changed to androgen-depleted medium (ADM) and the cells were treated with atorvastatin (10 μM) or celecoxib (10 μM) alone or in combination for 48 h. Survivin was determined by western blotting using an anti-survivin antibody (AB3610).

Due to the important role of IL-6 in prostate cancer progression (20–26), we determined the effect of atorvastatin and/or celecoxib on this cytokine. As shown in Fig. 2, culturing LNCaP cells in AD medium increased the level of IL-6. Treatment of the cells in AD medium with a combination of atorvastatin and celecoxib had a more potent effect for inhibiting the increase in IL-6 than either drug alone (Fig. 2). To further evaluate the role of IL-6 in apoptosis induction by atorvastatin and celecoxib, we determined whether addition of recombinant IL-6 may abrogate the stimulatory effect of atorvastatin and celecoxib on apoptosis. In these experiments, LNCaP cells were treated with a combination of atorvastatin and celecoxib in the presence or absence of recombinant IL-6. We found that addition of recombinant IL-6 significantly decreased the combined effect of atorvastatin and celecoxib on apoptosis in the LNCaP cells cultured in AD medium (Fig. 3). In additional experiments, the effects of atorvastatin and/or celecoxib on the level of survivin were determined using western blot analysis. We found that culturing the LNCaP cells in AD medium increased the level of survivin, and treatment of the cells with a combination of atorvastatin and celecoxib strongly inhibited the increase in the level of this protein (Fig. 4).

### Effect of atorvastatin and celecoxib treatment on the level of IL-6 and survivin in LNCaP tumors in castrated mice as they progress from a state of androgen-dependence to androgen-independence

**Figure 5:**
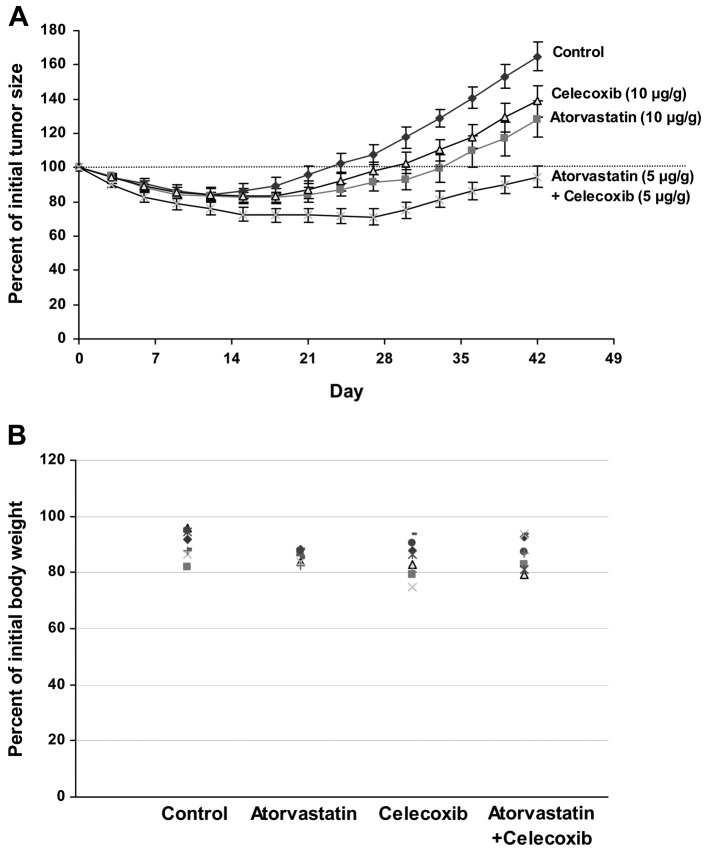
Effect of i.p. injections of atorvastatin or celecoxib alone or in combination on the progression and growth of androgen-dependent LNCaP xenograft prostate tumors to androgen-independence. Male SCID mice were injected subcutaneously with LNCaP cells in 50% Matrigel (2.0×10^6^ cells/0.1 ml). After 4–6 weeks, mice with LNCaP tumors (0.6–1.0 cm wide and 0.6–1.0 cm long) were surgically castrated. Castrated mice were injected i.p with atorvastatin (10 μg/g body weight/day), celecoxib (10 μg/g body weight/day) or a combination of atorvastatin (5 μg/g body weight/day) and celecoxib (5 μg/g body weight/day) for 42 days. Tumor size (length × width) and body weight were measured once every 3 days and expressed as a percentage of initial tumor size and percentage of initial body weight, respectively. (A) Growth curve of LNCaP tumors in each group. Each value represents the means ± SE from 8 mice (B) Individual body weight of mice after treatment for 42 days. [This figure was published in Cancer Prev Res 1: 114–124, 2010 (18)].

**Figure 6:**
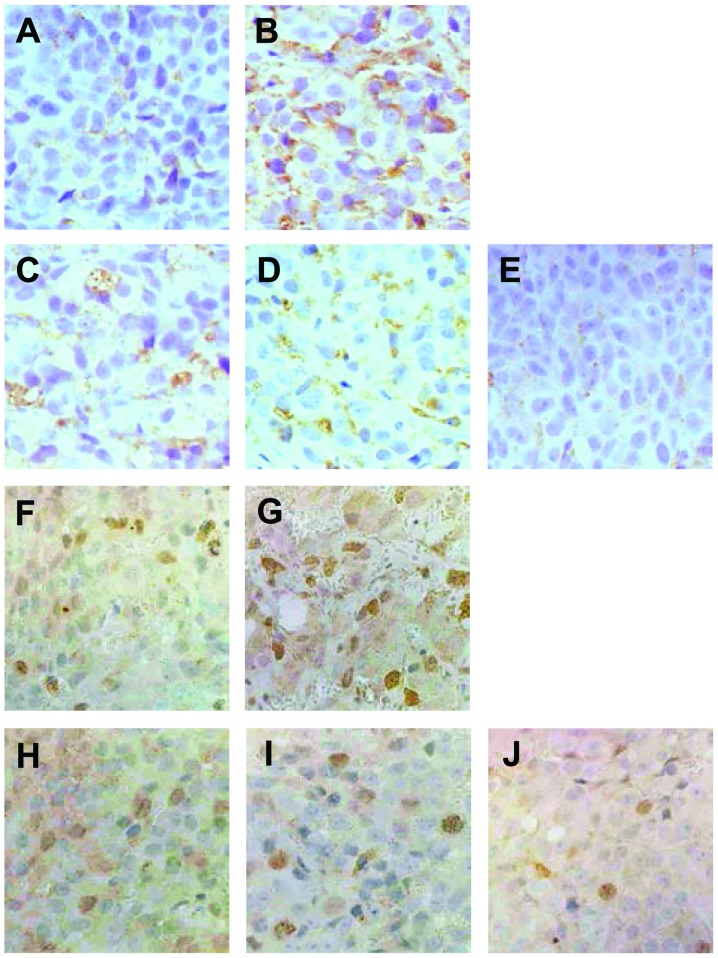
Immunohistochemistry of IL-6 and survivin in LNCaP tumors. Immunostaining using anti-IL-6 (AF-206-NA) or anti-survivin (AB3610) antibody was performed to determine the levels of IL-6 (A–E) and survivin (F–J) in LNCaP tumors. (A and F) LNCaP tumor before castration (androgen-dependent); (B and G) LNCaP tumor 42 days after castration and injected with vehicle once/day (androgen-independent); (C and H) LNCaP tumor from castrated mice treated with atorvastatin (10 μg/g body weight/day, i.p.) for 42 days; (D and I) LNCaP tumor from castrated mice treated with celecoxib (10 μg/g body weight/day, i.p.) for 42 days; (E and J) LNCaP tumor from castrated mice treated with the combination of the two drugs (5 μg/g body weight/day, i.p.) for 42 days. The data presented are representative of 8 animals/group.

To assess the effect of atorvastatin and celecoxib *in vivo*, we studied the effect of each agent alone and in combination on LNCaP xenograft tumors in SCID mice. We found that treatment with a combination of atorvastatin and celecoxib strongly inhibited the progression of androgen-dependent LNCaP tumors to androgen independence when compared to the vehicle treatment only or either agent alone (Fig. 5)(18). In the present study, the levels of IL-6 and survivin in LNCaP tumors as they became androgen-independent were determined using immunohistochemistry. We found that androgen-dependent LNCaP tumors (before castration) had low levels of IL-6 (Fig. 6A) and survivin (Fig. 6F). Increased levels of IL-6 (Fig. 6B) and survivin (Fig. 6G) were observed in androgen-independent tumors (42 days after castration). Treatment of the mice with atorvastatin alone caused a modest decrease in IL-6 (Fig. 6C) and survivin (Fig. 6H). Treatment of the mice with celecoxib alone also resulted in a moderate decrease in IL-6 (Fig. 6D) and survivin (Fig. 6I). However, treatment with a combination of celecoxib and atorvastatin resulted in a much stronger inhibition of the increased level of IL-6 (Fig. 6E) and survivin (Fig. 6J) in LNCaP tumors of the castrated mice.

## Discussion

In previous studies, we found that administration of atorvastatin and celecoxib in combination had a strong inhibitory effect on the progression of androgen-dependent LNCaP tumors to androgen-independence in castrated SCID mice, and combined drug treatment also inhibited the growth of androgen-independent prostate cancer PC-3 tumors (18,19). In the present study, we demonstrated that atorvastatin and celecoxib in combination strongly inhibited the formation of IL-6 in LNCaP cells grown in androgen-deficient medium as well as in LNCaP tumors as they became androgen-independent in castrated SCID mice. IL-6 is a pleiotropic cytokine originally identified as a regulator of immune and inflammatory responses (32), and evidence has been accumulating that IL-6 may also play an important role in prostate cancer progression to an androgen-independent state (24,25). Increased serum levels of IL-6 in prostate cancer patients were found to be associated with androgen-independence and metastatic disease (33,34), and overexpression of IL-6 was associated with protection of LNCaP cells from apoptosis during androgen depletion (26). Recent studies have shown that IL-6 can activate AR signaling in the absence of androgen (21–23).

In an *in vitro* study, we found that the level of IL-6 in LNCaP cells was increased after the cells were cultured in androgen-depleted medium. During androgen deprivation therapy, survival and proliferation of prostate cancer cells in the absence of androgen can occur and are important during the development of androgen-independence. Since IL-6 was shown to activate AR signaling (21–23), increased levels of IL-6 during androgen deprivation may enhance the survival and proliferation of prostate cancer cells. In the present study, we found that a combination of atorvastatin and celecoxib inhibited growth and stimulated apoptosis in LNCaP cells cultured in androgen-depleted (AD) medium, and their effects were associated with a decrease in IL-6 levels. Moreover, addition of recombinant IL-6 partially abrogated the effect of atorvastatin and celecoxib on apoptosis stimulation in LNCaP cells indicating that a decreased IL-6 level is important for the effect of atorvastatin and celecoxib. *In vivo*, we found that IL-6 expression was increased in androgen-independent LNCaP tumors. Treatment of the mice with atorvastatin or celecoxib alone resulted in a decrease in the level of IL-6 in the androgen-independent LNCaP tumors. The combination of atorvastatin and celecoxib caused a stronger inhibitory effect on the IL-6 level than either drug alone. The present study indicates that progression of androgen-dependent LNCaP tumors to androgen independence is associated with an increase in IL-6 and that treatment with atorvastatin and celecoxib inhibited the increased tumor level of IL-6.

Survivin is a member of the inhibitor of apoptosis (IAP) gene family, and is involved in the control of mitotic progression and inhibition of apoptosis (35). In prostate cancer, overexpression of survivin has been associated with increased cancer aggressiveness and reduced patient survival (36). Survivin is a downstream target of the signal transducer and activator of transcription 3 (Stat3) that can be activated by IL-6 in prostate cancer (21,26,37). In the present study, we found that treatment with atorvastatin and celecoxib in combination markedly decreased the levels of IL-6 and survivin in cultured LNCaP cells in AD medium as well as in LNCaP xenograft tumors in castrated SCID mice. Our studies indicate that the effects of atorvastatin in combination with celecoxib on prostate cancer LNCaP cells are associated with inhibition of the IL-6 signaling pathway.

In summary, we demonstrated in the present study that atorvastatin and celecoxib in combination strongly decreased the levels of IL-6 and survivin in cultured LNCaP cells grown in AD medium and in LNCaP xenograft tumors in castrated SCID mice. Addition of IL-6 partially abrogated the apoptosis-inducing effect of atorvastatin and celecoxib in LNCaP cells. This result indicates that the effect of a combined treatment of atorvastatin and celecoxib on apoptosis is mediated, at least in part, by inhibition of the formation of IL-6. The present study suggests that the IL-6 signaling pathway may be a useful target for the prevention of androgen-dependent prostate cancer progression to androgen-independence.
